# Evolutionary Multiobjective Query Workload Optimization of Cloud Data Warehouses

**DOI:** 10.1155/2014/435254

**Published:** 2014-04-29

**Authors:** Tansel Dokeroglu, Seyyit Alper Sert, Muhammet Serkan Cinar

**Affiliations:** ^1^METU Computer Engineering Department, Cankaya, 06800 Ankara, Turkey; ^2^Hacettepe University Computer Engineering Department, Beytepe, 06800 Ankara, Turkey

## Abstract

With the advent of Cloud databases, query optimizers need to find paretooptimal solutions in terms of response time and monetary cost. Our novel approach minimizes both objectives by deploying alternative virtual resources and query plans making use of the virtual resource elasticity of the Cloud. We propose an exact multiobjective branch-and-bound and a robust multiobjective genetic algorithm for the optimization of distributed data warehouse query workloads on the Cloud. In order to investigate the effectiveness of our approach, we incorporate the devised algorithms into a prototype system. Finally, through several experiments that we have conducted with different workloads and virtual resource configurations, we conclude remarkable findings of alternative deployments as well as the advantages and disadvantages of the multiobjective algorithms we propose.

## 1. Introduction

Cloud computing has emerged as a new computation paradigm that builds elastic and scalable software systems. Vendors such as Amazon, Google, Microsoft, and Salesforce offer several options for computing infrastructures, platforms, and software systems [1–4] and supply highly scalable database services with simplified interfaces and the goal of reducing the total cost of ownership [5–7]. Users pay all costs associated with hosting and querying their data where database-as-a-service providers present different choices to tradeoff price and performance to increase the satisfaction of the customers and optimize the overall performance [8, 9]. Recently, extensive academic and commercial research is being done to construct self-tuned, efficient, and resource-economic Cloud database services that protect the benefits of both the customers and the vendors [10–13].

**Figure 1:**
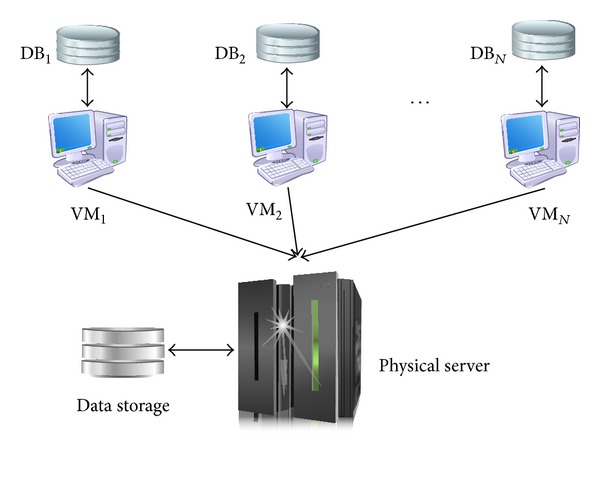
Virtualization of resources on a server.

Virtualization is being exploited to simplify the administration of physical machines and accomplish efficient systems and it is the main enabling technology of Cloud computing that provides the illusion of infinite resources in many respects [14]. The perception of hardware and software resources is decoupled from the actual implementation and the virtual resources perceived by applications are mapped to real physical resources. Through mapping virtual resources to physical ones as needed, the virtualization can be used by several databases that are located on physical servers to share and change the allocation of resources according to query workloads [15] (see Figure 1). This capability of virtualization provides efficient Cloud databases where each virtual machine (VM) has its own operating system and thinks that it is using dedicated resources (CPU, main memory, network bandwidth, I/O, etc.), whereas in reality the physical resources are shared among by using a VM monitor (VMM) that controls the allocation of resources [16–19].

In addition to providing efficient queries in accordance with the service level agreements, contemporary relational Cloud database management systems need to optimize a multicriteria problem that the overall cost of hardware ownership price is to be minimized. More specifically, the problem can be stated as follows.

Given a budget constraint and a query workload, how can the virtual resources of the Cloud (CPU, main memory, network bandwidth, etc.) be allocated to virtual machines, each having a part of a distributed database, that the best overall query performance can be achieved with minimum pricing?

In this study, we have developed a framework to provide (near-)optimal virtual resource allocations with respect to the overall cost of hardware ownership price and a good tradeoff between the efficiency and the overall cost of a database is ensured. Our framework produces cost-efficient design alternatives (virtual resource configuration and query plans) and recommends them to decision makers. A budgetary constraint can be a more important criterion for a consumer, whereas the response time of the queries is more crucial for another [20]. Therefore, in order to fully realize the potential of the Cloud, alternative query plans are executed with well configured virtual resources instead of only optimizing single query plans on statically designed virtual resources [21]. This means that instead of designing the database over standard VMs, we have configured the virtual resources, which indicates that CPU usage and RAM can be a crucial point for a data warehouse workload, whereas network or I/O bandwidth is more important for another.

**Figure 2:**
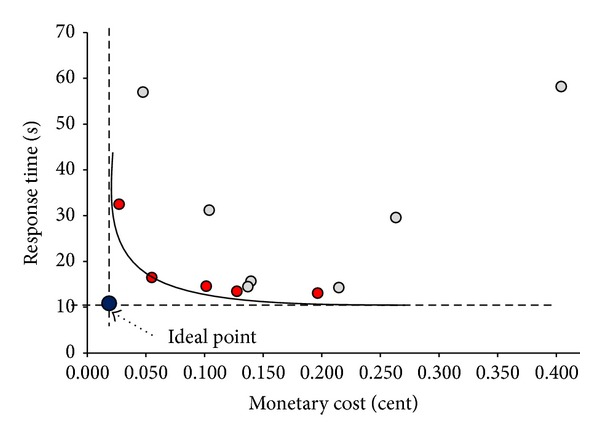
Paretooptimal curve for the response time and monetary cost of TPC-H Q3 with different virtual resource configurations and query plans.

In this part, we give a scenario to explain the multiobjective problem in more detail. Consider a distributed TPC-H decision support database where all of its tables are located on different VMs. When we execute TPC-H query 3 with two different query plans (QP_1_ and QP_2_ given in Appendix) and with alternative virtual resource allocations, we obtain different results. The results of this experiment are presented in Tables 1 and 2. As it can be seen, the configuration of VMs with 4 × 2 Ghz CPU, 8 GB RAM, and 300 Mbps network bandwidth and with QP_1_ is observed to be the best performing platform; however, its monetary price is one of the most expensive alternatives. The configuration of 1 × 2 Ghz CPU, 768 MB RAM, and 100 Mbps network bandwidth and with QP_1_ has a response time, that is, only 25.9% slower but 72.0% cheaper. The paretooptimal visualization of the solutions can be seen in Figure 2. Looking at the results, the cheapest VM configuration is not the worst and the most expensive configuration is not the best solution in accordance with both objectives. Instead, alternatives chosen according to virtual resource demands provide better paretooptimal solutions. For example, QP_1_ requests more network resources, whereas QP_2_ spends more main memory. In this case, QP_1_ can be a better way to execute a query where the network bandwidth is high and cheap.

In order to investigate the effectiveness of our approach, we incorporate the devised framework into a prototype system for evaluation and instantiate it with a simple heuristic algorithm (SHA), an exact solution method, branch-and-bound (MOBB), and a soft computing method multiobjective genetic algorithm (MOGA). Finally, through several experiments that we have conducted with the prototype elastic virtual resource deployment optimizer on TPC-H query workloads, we conclude remarkable results of the space of alternative deployments as well as the advantages and disadvantages of the multiobjective optimization algorithms.

The remainder of this paper is organized as follows. In Section 2, we provide information about the related studies. In Section 3, we give mathematical multiobjective query optimization problem formulation. In Section 4, infrastructure and pricing scheme parameters of the Cloud are given.  Section 5 proposes our simple heuristic (SHA), branch-and-bound (MOBB), and genetic algorithms (MOGA).  Section 6 defines our experimental environment and the setup of the selected TPC-H query workloads and presents the results of the experiments. In Section 7, we give our concluding remarks.

## 2. Related Work

In this section, we summarize some of the studies related to our work. There has been a lot of research related to the Cloud, but relatively there is no approach like ours that is concerned with both the optimization of the total ownership price and the performance of the queries by taking into account alternative virtual resource allocation and query plans.

Distributed databases are considered to be the first representatives of the Cloud databases. Therefore, we first analyzed Mariposa, an early distributed database system that implements an economic paradigm to solve many drawbacks of wide-area network cost-based optimizers [22]. In Mariposa, clients and servers have an account in a network bank and users allocate a budget to each of their queries. The processing mechanism aims to service the query in the limited budget by executing portions of it on various sites. The latter place bids for the execution of query parts and the bids are collected in query brokers. The decision of selecting the most appropriate bids is delegated to the user. A series of similar works have been proposed for the solution of the problem [23, 24]. Papadimitriou and Yannakakis [25] showed that Mariposa's greedy heuristic can be far from the optimum solution and proposed that the optimum cost-delay tradeoff (Pareto) curve in Mariposa's framework can be approximated fast within any desired accuracy. They also present a polynomial algorithm for the general multiobjective query optimization problem, which approximates the optimum cost-delay tradeoff.

An advisor automatically configures a set of VMs for database workloads where the advisor requests domain knowledge in Soror's study [15]. Although his approach concerns with the efficient allocation of the VMs, it does not optimize the total ownership price of the system. Recently, efficient cost models have been proposed in the Cloud for scheduling of dataflows with regard to monetary cost and/or completion time [12] and cost amortization of data structures to ensure the economic viability of the provider [13], particularly for self-tuned caching [11] and for a real-life astronomy application using the Amazon Cloud [26].

New cost models that fit into the pay-as-you-go paradigm of Cloud computing are introduced in [10]. These cost models achieve a multiobjective optimization of the view materialization versus CPU power consumption problem under budget constraints. It is shown that Cloud view materialization is always desirable. Koutris et al. [27] built a theoretical foundation, the first one towards a practical design and implementation of a pricing system. They present a formal framework for query-based pricing. Central to this framework are the notions of arbitrage-free and discount-free pricing.

In [28], the cost performance tradeoffs of different execution and resource provisioning plans have been simulated, showing that by provisioning the right amount of storage and computing resources, cost can be reduced significantly. The performance of three workflow applications with different I/O, memory, and CPU requirements has also been compared on Amazon EC2 and a typical high-performance cluster (HPC) to identify what applications achieve the best performance in the Cloud at the lowest cost [26].

Recent research takes interest in various aspects of database and decision support technologies in the Cloud. Different studies investigate the storage and processing of structured data [29], the optimization of join queries, and how to support analysis operations such as aggregation [30]. Cloud data warehousing and OLAP systems also raise various problems related to storage and query performance [31]. Adaptations of these technologies to the Cloud are addressed in [32] or the calculation of OLAP cuboids using the MapReduce runtime environment [33].

In [34], a virtual-machine provisioning policy based on marginal cost and revenue functions is proposed. For each Cloud customer there exists a budget as a function of the execution time of the tasks that are submitted. Knowledge of this function, combined with the machine-hour cost, allows for educated decisions regarding the amount of virtual resources allocated per customer in the context of an IaaS-Cloud, an answer to the question of exactly how many VMs a consumer should request from a Cloud within a budget.

In [35], a cost-aware provisioning system for Cloud applications that can optimize either the rental cost for provisioning a certain capacity or the transition cost of reconfiguring an application's current capacity is proposed. The system exploits both replication and migration to dynamically provision capacity and uses an integer linear program formulation to optimize cost.

There has been a substantial amount of work on the problem of tuning database system configurations for specific workloads or execution environments [36] and on the problem of making database systems more flexible and adaptive in their use of computing resources [37, 38]. In our study we are tuning the virtual resources to a database system, rather than tuning the database system for a given resource setting. Also our study optimizes the objectives of minimum money consumption and maximum benefit from the virtual resources and optimizes this with an efficient multiobjective genetic algorithm. In summary, our study focuses on the elasticity of Cloud resources and produces multiple resource deployment plans with alternative query plans for a set of queries in a batch, enabling the user to select the desired tradeoff with efficient cost models. To the best of our knowledge, no resource deployment processing system deals with the concept of elasticity and cost-efficiency on relational Cloud databases like our system.

## 3. Multiobjective Query Optimization Problem Formulation

In this part, we first give the definitions of some main terms that are used in the study to provide the reader a better understanding and later we present the mathematical representation of our multiobjective query optimization problem of a data warehouse workload on the Cloud.

*Virtual Machine (VM)*. VM is software that emulates the architecture and functions of a real computer. The number of its processors and main memory can be changed through virtualization. 

*Distributed Data Warehouse*. Distributed data warehouse is a TPC-H decision support data warehouse that is distributed over a set of VMs on a network. VMs communicate with each other by paying a cost required by Cloud vendor. 

*Workload (W)*. Workload is a set of queries that are submitted as a batch to a distributed data warehouse. Our data warehouse is a distributed database so that the tasks of the queries are sent to the related VM and its results are received by other VMs to join the data and give a result. 

*Response Time*. Response time is the time that has elapsed between the submission of the queries and obtaining of the results. 

*Total Execution Time*. Total execution time is the sum of the time spent by the CPUs of the VMs and the time period during the data transmission over the network. 

*Monetary Cost*. Monetary cost of a Cloud data warehouse workload (*C*_total_) includes renting the resources to run the database. These resources are mainly data storage (*C*_storage_) processing time of the VMs (*C*_ comp _) and the sum of data transfer cost (*C*_comm_) [10]:

(1) $$\begin{array}{c} C_{\text{total}}=C_{\text{storage}}+C_{\text{ comp }}+C_{\text{comm}}. \end{array}$$

Data storage cost, *C*_storage_, depends on the size of the data (including the structures such as indexes and replications) and its storage time. Processing time of the VMs, *C*_ comp _, is the total price for CPU usage. During the execution of the queries, different VM configurations can be used and the configuration of a VM (RAM, number of CPUs, etc.) is flexible in accordance with the resources used. Micro, small, large, and extra-large are some of the configurations provided by the Cloud vendors at various prices [3]. Data transfer cost, *C*_comm_, is related to the amount of data migrated between sites and the pricing model applied by the Cloud provider. 

**Figure 3:**
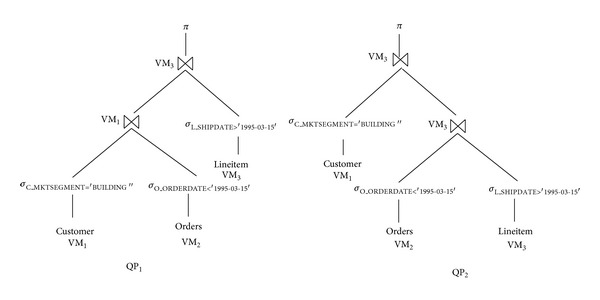
Alternative QPs for TPC-H Q3 query.

*Alternative Query Plans (QPs)*. QPs provide different ways for executing a query. In Figure 3, we can see two different QPs of TPC-H query Q3. QP_1_ first joins the customer and orders relations, whereas QP_2_ joins the orders and lineitem relations first. Alternative QPs can take advantage of different ways of executing the same query; thus, cheaper resources can reduce the total price of a query while increasing the response time. This elasticity provides new opportunities for the solution of our multiobjective problem.

The formulation of the problem consists of two parts: the monetary cost and the response time of the query workloads that will work on the selected VMs with the alternative QPs of the queries.

The monetary cost is calculated in accordance with (1) and the response time of the query workloads is calculated with the parameters and statistics used by query optimizers. The main goal of the problem is to minimize

(2) $$\begin{array}{c} F\left(x\right)=\left\{\text{Resp}\_\text{time}\left(x\right),\text{Total}\_\text{cost}\left(x\right)\right\}, \end{array}$$

where *x* denotes a solution vector consisting of a set of VMs, QPs of *m* queries in workload *W*_*k*_ that will be executed on selected VMs, and the following network:

(3) $$\begin{array}{c} x=\left\{\left\{\text{V}{\text{M}}_{1},\ldots ,\text{V}{\text{M}}_{n}\right\},\left\{{\text{QP}}_{1},\ldots ,{\text{QP}}_{m}\right\},{\text{Network}}_{i}\right\}. \end{array}$$

There are *n* VMs with independent DBMS and each VM has a set of processors and a main memory. Each DBMS has a workload that consists of a set of queries. *W*_*i*_ represents the *i*th workload. The resources to be deployed to VMs are CPU and main memory.

M_Cost(*W*_*k*_, *x*_*i*_) is the total monetary cost of workload *W*_*k*_ for the solution vector *x*_*i*_.

Resp_Time(*W*_*k*_, *x*_*i*_) is the response time of workload *W*_*k*_ with the virtual resource and QP settings given in solution *x*_*i*_.

The main goal is to obtain the paretooptimal set of solutions such that the overall workload cost is minimized. The overall multiobjective objective function (finding solutions closer to the ideal point given in Figure 2) is represented in the following:

(4) F(xi)=min⁡(Resp_time(xi)−Best_time(x))2+(M_cost(xi)−Best_cost(x))2,

where Best_time(*x*) is the best response time and Best_cost(*x*) is the minimal monetary cost for workload *W*_*k*_ with solution vector *x*. 

*Response Time Cost Model*. In order to measure the response time of a workload with a configuration of VMs, we developed a response time based cost model [21, 39] that uses bushy-plan trees [40]. The cost model depends on the statistics of the database catalog. The main parameters used in the cost model are shown in Table 3.

Using the number of pages as a parameter, the response time taken by a query is calculated as

(5) Resp_time=(TCPU∗seq_#insts)+(TI/O∗seq_#I/Os)+(TMSG∗seq_#msgs)+(TTR∗seq_#bytes).

The network communication time of transferring an intermediate query result from one site to another is calculated as

(6) $$\begin{array}{c} \text{CT}\left(\#\text{pages}\right)=T_{\text{MSG}}+\left(T_{\text{TR}}*\#\text{pages}\right). \end{array}$$

## 4. Infrastructure and Pricing Scheme Parameters of the Cloud

In this section, we describe the infrastructure details of the Cloud database and the pricing scheme that we have used during the optimizations. Each customer requests queries from the Cloud by using Internet and contacts with the aggregate node. The aggregate node distributes the query to the appropriate VMs. The Cloud infrastructure provides unlimited amount of storage space, CPU nodes, RAM, and very high speed intra-Cloud networking. All the resources of the Cloud are assumed to be on a network. The CPU nodes, RAM, and I/O bandwidth of each VM are different and can be deployed by using VM monitors (VMM) in milliseconds [14]. The storage system is based on a clustered file system where the disk blocks are stored close to the CPU nodes accessing them. I/O bandwidth of the storage is divided evenly to the VMs (that may have multiple cores up to 8).

There are many Cloud service providers (CSP) in the market and they offer different pricing schema for the services they provide. Different pricing schema of Cloud server providers can be opportunities for customers in accordance with the tasks they want to complete. In our study, we will use a pricing scheme that is similar to Windows Azure [3]. VM configurations such as extra small (XS), small (S), and medium (M) are provided by the Cloud service provider. The detailed information of VM configurations can be seen in Table 4. The cost for a small VM (1 GHz CPU, 768 MB RAM) is $0.02/hr, whereas for A7 (8 × 1.6 GHz CPU, 56 GB RAM) is $1.64/hr.

Data storage is also billed by the Cloud service providers. In our model, monthly storage price is used. During our experiments, the data storage price was constant for all the queries. Therefore, we do not add this parameter to our overall cost. The detailed information of database storage prices can be seen in Table 5.

Most of the Cloud providers do not charge for the data transfers in a private Cloud but the data that leaves the Cloud and the bandwidth of the intra-Cloud network can reach up to 10 Gbps. In order to make our problem more interesting and handle this dimension of the optimization, we have located our VMs on a virtual switch. Different bandwidth networks can be chosen and the pricing scheme changes in this communication infrastructure. The pricing we have used for the network layer is given in Table 6. The bandwidth of the network is increased from10 Mbps up to 200 Mbps during the experiments.

## 5. Proposed Algorithms

In this section, we propose three algorithms for the solution of the problem: simple heuristic algorithm (SHA), an exact algorithm branch-and-bound which finds the paretooptimal solutions, and a multiobjective genetic algorithm (MOGA).

### 5.1. Simple Heuristic Algorithm (SHA)

In SHA, we have ranked the VMs in accordance with the frequency of the join operations. For example, with the database configuration we have studied, the first one was the VM that most of the join operations took place and we have assigned the best configuration to this VM. For the rest of the VMs we have assigned configurations with decreasing prices. The effects of all network types and QPs are evaluated on these configurations of the VMs. This algorithm is developed to provide us a test environment to evaluate the performance of other proposed algorithms, MOBB and MOGA.

### 5.2. Multiobjective Optimization of Cloud Database Configuration Using Branch-and-Bound Algorithm (MOBB)

Multiobjective branch-and-bound algorithm (MOBB) is an exhaustive optimization algorithm. It enumerates all candidate solutions, where fruitless subsets of candidates are discarded, by using upper and lower estimated bounds of the problem instance being optimized [41]. MOBB starts searching with* null* initial values indicating that no QP has yet been selected for any queries with the current VM configuration. Later, QPs are assigned to selected VM configuration. At each level of the tree, one additional QP is assigned to the query workload. This procedure is repeated for every VM configuration.

**Figure 4:**
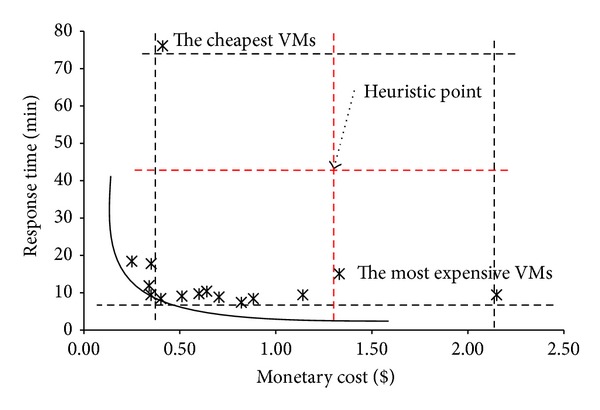
Proposed heuristic value for MOBB algorithm.

We define two initial upper bounds for MOBB. The minimum monetary cost is the running time of VMs that execute the queries in a workload of queries. The response time is the finishing time of the workload with the given VM configuration. In order to estimate a lower bound, different heuristic functions can be used. The heuristic we proposed here is reasonable and performs well during the optimization process. We will explain the heuristic with a scenario. In Figure 4 we can see the results of a sample multiobjective query workload optimization. The best response time and the minimum monetary cost values are defined and marked on the Figure. We can obtain these values with the most expensive and the cheapest VM configurations easily. Hereby, we propose a heuristic point (marked as* heuristic point* on the Figure), that is, the center of the square constructed by the response time and monetary costs of the most expensive and the cheapest VM configurations. If the response time of a workload falls above heuristic point or if the monetary cost is at the right-hand side of heuristic point on the Figure then it is pruned according to our heuristic.

Table 7 gives us an execution order of a sample workload* W*. QP_1_ denotes the first query execution plan of a query and 〈QP_1_, QP_1_,…, QP_2_〉 is the sequence of submitted queries in a workload. The first query is executed with query plan QP_1_, the second query is executed with query plan QP_1_, and the last query is executed with query plan QP_2_. The executions in the table start with query execution plan of query 1 and two null queries. The final solution is the state with no* null* values. After finding the best and worst response times with the most expensive and the cheapest VM configurations, we set our heuristic point as monetary cost = $1.2 and response time = 50 min.

The execution starts by assigning the queries to the current VM configuration. The response time and the monetary cost of the first query are calculated with its three different QPs. The first QP is in the acceptable bounds (monetary cost = $0.5 and response time = 10 min.) but the other two QPs exceed the limits. The second one is more expensive than the heuristic value ($1.3) and the third one is slower than the heuristic value (55 min.). Therefore, they are pruned. In the second phase, we assign the QPs of the second query. They are within the limits of the heuristic value and at the last phase we add the third query. They do not exceed the limits of the heuristic value and they become solutions. Pseudocode of our MOBB algorithm is given in Algorithm 1.

### 5.3. Multiobjective Optimization of Cloud Database Configuration Using Genetic Algorithm (MOGA)

The principles of applying natural evolution to optimization problems were first described in [42, 43]. The GA theory has been further developed and GAs have become very powerful tools for solving search and optimization problems [44–46]. GAs are based on the principle of genetics and evolution and have been frequently used to solve many NP-complete problems. GAs use a computational model that simulates the natural processes of selection and evolution. Individuals with better quality have more chance to survive, to reproduce, and to pass their genetic characteristics to future generations. Each potential solution in the search space is considered as an individual and is represented by strings called chromosomes. Genes are the atomic parts of chromosomes and codify a specific characteristic. Chromosomes are encoded in different ways for each application. A random population is generated in the first step of the algorithm and by applying selection, crossover, and mutation operations iteratively new generations are created [47]. The individual having the best fitness value in the population is returned as the solution of the problem.  Algorithm 2 gives the details of GA used in MOGA system.

**Figure 5:**
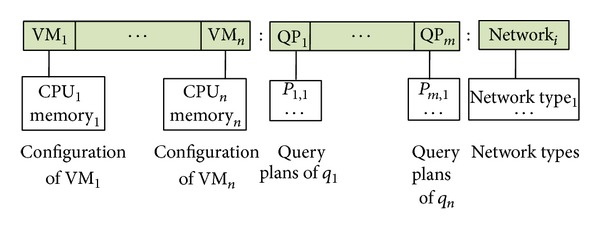
Chromosome structure for the proposed multiobjective genetic algorithm that consists of the virtual machines, the query plans, and the network layer.

Multiobjective query optimization problem can be modeled by evolutionary methods. A chromosome corresponds to a solution instance including a set of relational Cloud database QPs.  Figure 5 shows the chromosome structure of a solution instance. The chromosome consists of three parts; leftmost segment represents the configuration of the VMs. Middle segment is the set of QPs for the queries in the workload. Rightmost part gene represents the selected network layer of the solution vector.

We have defined three operators for the solution of GA model.

**Figure 6:**
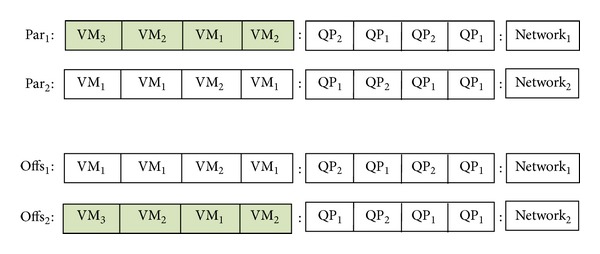
Global crossover operator for the multiobjective optimization of query workloads.

*Crossover Operator*. The operator uses two parents that are selected from the population by a selection method. We have proposed two types of crossover operators,* global* and* local*. Global crossover operator swaps VM, QP, or network part of two selected chromosomes with the same counter chromosome. Below we can see two parents and their VM parts are exchanged to provide two new chromosomes. Details can be seen in Figure 6. Consider

(7) par1={{VM3,VM2,VM1,VM2}, {QP2,QP1,QP2,QP1},Network1}par2={{VM1,VM1,VM2,VM1}, {QP1,QP2,QP1,QP1},Network2}.

New offspring (offs) are

(8) offs1={{VM1,VM1,VM2,VM1}, {QP2,QP1,QP2,QP1},Network1}offs2={{VM3,VM2,VM1,VM2}, {QP1,QP2,QP1,QP1},Network2}.

**Figure 7:**
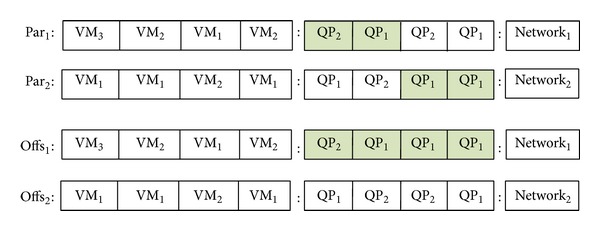
Local crossover operator for the multiobjective optimization of query workloads.

The local crossover operator works on the VM and QP segments of the chromosome by dividing the parents and exchanging the segments with each other.  Figure 7 gives an example of local crossover that divides the QPs of the chromosomes and exchanges to generate new offspring. 

**Figure 8:**
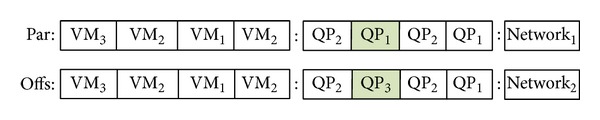
Mutation operator for the multiobjective optimization of query workloads.

*Mutation Operator*. Mutation operator changes a randomly selected gene of a chromosome. In our chromosome structure this operator can act on any of the segments. Only a gene is replaced at every mutation process.  Figure 8 shows how a mutation operator changes a QP in a chromosome. 

*Fitness Calculation*. Multiobjective fitness evaluation does not produce a single solution vector. Therefore, we have selected the nondominant individuals in the population as the resulting solution set. The fitness of the individuals is evaluated in accordance with (4).

Parameters of MOGA are as follows:

- *population size:* total number of chromosomes (individuals) in each generation;
- *number of generations:* each iteration of a GA that a number of crossovers and mutations are applied;
- *maximum number of genes to transfer:* maximum length of the crossed segment in segmented crossover operation and maximum number of genes transferred in a multiple-point crossover operation;
- *minimum number of genes to transfer:* minimum length of the crossed segment in segmented crossover operation and minimum number of genes transferred in a multiple-point crossover operation.
- *selection type (tournament): r* chromosomes (*r* is the tournament size) are selected from the population, and the chromosome with the best fitness value is chosen for the next generation from the* r*-element group; this process is repeated as many times as the population size of the next generation;
- *tournament size:* number of individuals entering a selection in tournament selection technique;
- *truncate ratio:* ratio of the best individuals, which are sorted according to their fitness values, used for producing the next generation;
- *mutation ratio:* probability of mutations in a single gene.

The results of the experiments with SHA, MOBB, and MOGA are presented in Section 6.

## 6. Experimental Evaluation

In this section, we describe our experimental environment, the setup of the selected TPC-H query workloads, VMM Hyper-V, and parameter settings for MOGA and present the results of the experiments we have obtained with multiobjective simple heuristic algorithm (SHA), branch-and-bound (MOBB), and multiobjective genetic algorithm (MOGA). The VM and network configurations are first optimized by the algorithms and later we have run the workloads on a real Cloud database to see the real results and measure the correctness of our algorithms.

### 6.1. Experimental Environment

**Figure 9:**
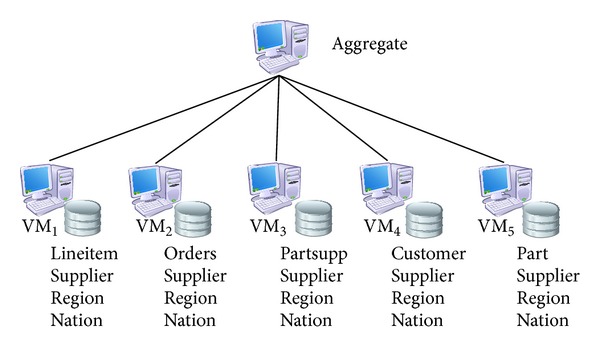
Architecture of the proposed system.

We have performed our experiments on a private Cloud server: 4U DELL PowerEdge R910 having 32 (64 with Hyper Threading) cores and each core is Intel Xeon E7-4820 with a total of 2.00 Ghz processing power. Server has 128 GB DDR3 1600 Mhz virtualised memory and Broadcom Nextreme II 5709 1 Gbps NICs. Operating system is Windows Server 2012 Standard Edition and as guest operating systems Windows Server 2008 R2 SP2 Enterprise Edition is used and on top of guest operating system, SQL Server 2012 Enterprise Edition Service Pack 1 is implemented as the database server. Windows Hyper-V 3.0 is used as virtualization platform. Network page size was 4 KByte during the experiments. The configuration of distributed data warehouse infrastructure we have used during the experiments is given in Figure 9. The resources (CPUs, main memory, and network bandwidth) of the VMs are changed according to the optimized solutions. VM* aggregate* is used to submit the workloads and obtain the results.

Hyper-V, known as Windows Server Virtualization, is a native hypervisor that enables platform virtualization on x86-64 systems. Hyper-V implements isolation of VMs in terms of a partition which is a logical unit of isolation, supported by the hypervisor, where each guest operating system executes. A hypervisor instance has to have at least one parent partition, running a supported version of Windows Server (2008, 2008 R2, or 2012). The virtualization stack runs in the parent partition and has direct access to the hardware devices. The parent partition later creates the child partitions which host the guest OSs.

### 6.2. TPC-H Workloads

A TPC-H database of size 10 GB is used during the experiments. The TPC-H database has 8 relations: lineitem (8,145 MB), orders (1,757 MB), partsupp (1,236 MB), part (290 MB), customer (256 MB), supplier (2 MB), region (0,008 MB), and nation (0,008 MB). The tables are assumed to locate at 5 different VMs. By replicating the small tables, nation, supplier, and region, we aimed to obtain a better performance.

We have used three different workloads that consist of TPC-H queries. Our purpose was to test the proposed algorithms under diverse workloads, in terms of the response time of query execution times and total cost of ownership. Each workload consists of 10 to 15 different TPC-H queries with predicates. Workload 1 has first 10 TPC-H queries, workload 2 has queries with smaller relations, and workload 3 has queries with larger relations and joined operations. For each query we have selected two QPs (including the best QP) on the average during the experiments. QPs can have more than a single task and these tasks can have dependencies with the other tasks to complete a query. Selected queries for the workloads are given in Table 8.

**Figure 10:**
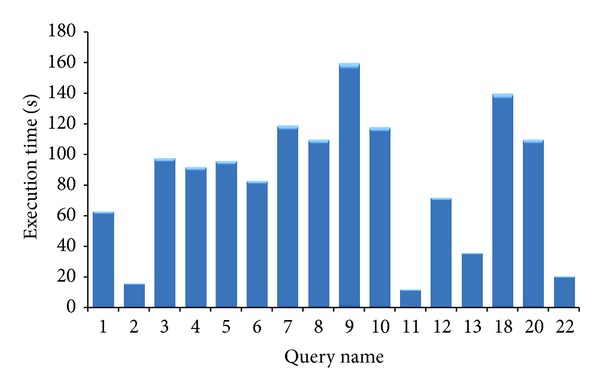
Response time of sample queries with the highest performance configuration VM settings and network bandwidths.

In Figure 10, we have presented the response time of the selected TPC-H queries we have used during the experiments. These response times are obtained with the highest configuration of VMs (XL) and network bandwidth (200 Mbps).

### 6.3. Parameter Settings for Multiobjective Genetic Algorithm

**Figure 11:**
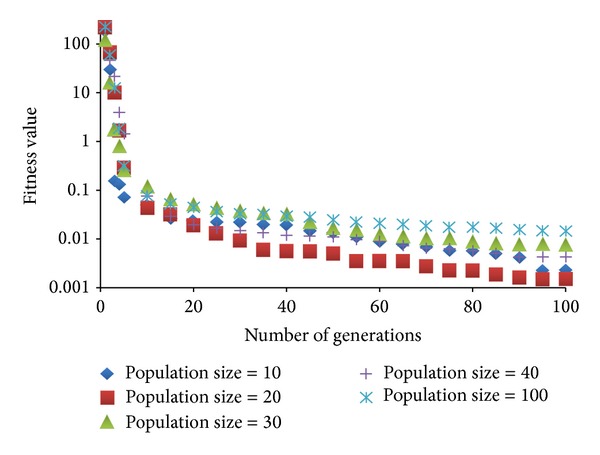
Average fitness value of different populations during generations.

Population size and the number of generations of a genetic algorithm are the most important parameters that must be well tuned to obtain (near-)optimal solutions during the optimization. Larger number of individuals and generations explore the search space more effectively. On the other hand, this may bring very long optimization times. In order to diminish the effect of this drawback we have performed some experiments with changing number of population sizes and generations. In Figure 11, we give the performance details of MOGA with different population sizes (10 to 100) and number of generations (10 to 100) for workload 1. The figure gives the average fitness value of populations during the generations. As it can be seen, MOGA almost converges after 100 generations and continues to improve itself slightly after this point. Although population size 10 seems to perform as the best option, population size 40 produces individuals that are more close to the ideal point that we aim to find.

**Figure 12:**
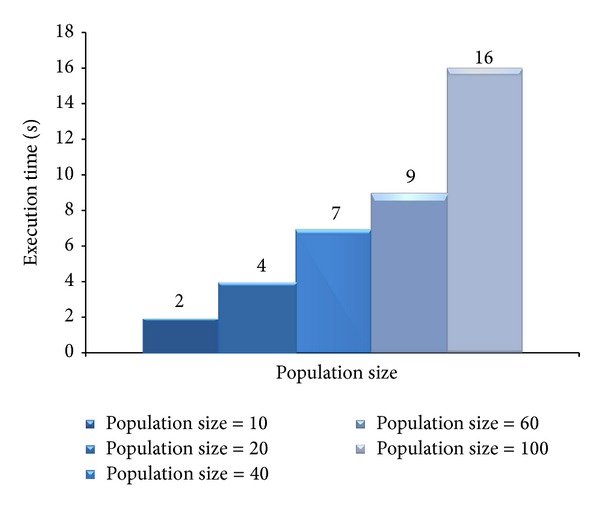
Average fitness value of different populations during generations.

Figure 12 gives the optimization times of MOGA with increasing number of populations. The optimization time of MOGA increases in accordance with the number of individuals in the population. For 10 individuals optimization time is 3 seconds and for 100 individuals it is 60 seconds. We have selected 40 individuals and 100 generations as our (near-)optimal parameters for the optimizations. These values provide good solutions for moderate size workloads such as ours.

**Figure 13:**
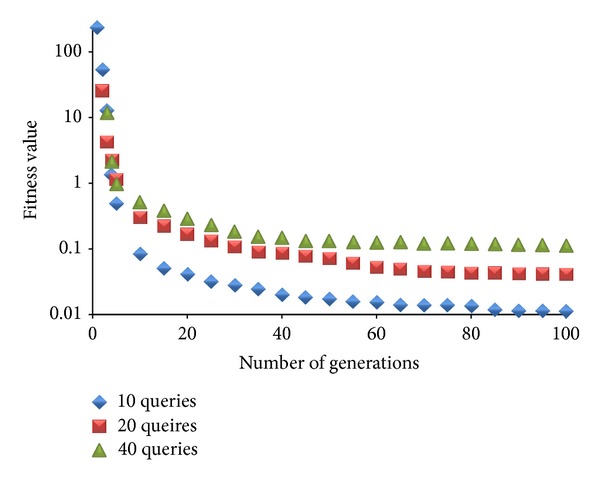
Average fitness values of 40 individuals and 100 generations for increasing number of submitted queries.

In Figure 13, we have analyzed the effect of increasing number of submitted queries for MOGA with 40 individuals and 100 generations. There are three sets of queries (10, 20, and 40). It can be seen that increasing the number of submitted queries decreases the average fitness quality of the population. With 10 queries, we can obtain solutions below 0.01 fitness value. For 20 and 40 queries the solution quality increases to 0.04 and 0.11, respectively. Although the average fitness values get worse as the number of submitted queries increases, the values are still not more than 0.11, which is very efficient. MOGA improves the quality of the individuals in accordance with the objective response time and monetary costs.  Table 9 shows our parameters settings used for MOGA. Crossover and mutation ratio are the values proposed by Holland [42]. Tournament is a very effective selection mechanism we have applied in our previous studies [44].

### 6.4. Experiments with the Workloads

In this part, we have performed experiments with the three TPC-H workloads we have defined previously. These workloads are first optimized with SHA, MOBB, and MOGA algorithms. Later, selected solutions are executed in our Cloud database environment to verify the correctness of our approach. There are 15 alternative virtual resource configurations in these tests: 3 SHA, 5 MOBB, 5 MOGA, the highest performance VM configuration, and the cheapest VM configuration. The last three solutions are used to measure the effectiveness of other solutions. The workloads are executed 10 times with the selected VM configurations and the average values are used.

**Figure 14:**
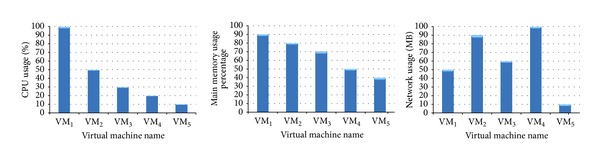
CPU, network, and memory consumption of the virtual machines.

Figure 14 shows snapshots of CPU, network, and memory consumptions of WMs during the execution of workload W_1_, respectively. As it can be seen WM_1_ demands the largest CPU resource and memory usage and VM_2_ and VM_4_ ship larger amounts of data. These snapshots are provided to give an idea about the resource demands of VMs during the execution of a workload.

The results of experiments with workloads 1, 2, and 3 are as follows.

**Figure 15:**
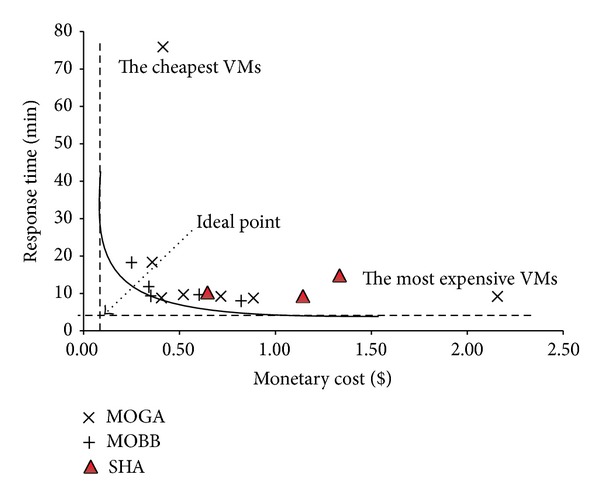
Proposed paretooptimal solutions for workload 1 by SHA, MOBB, and MOGA algorithms.

**Figure 16:**
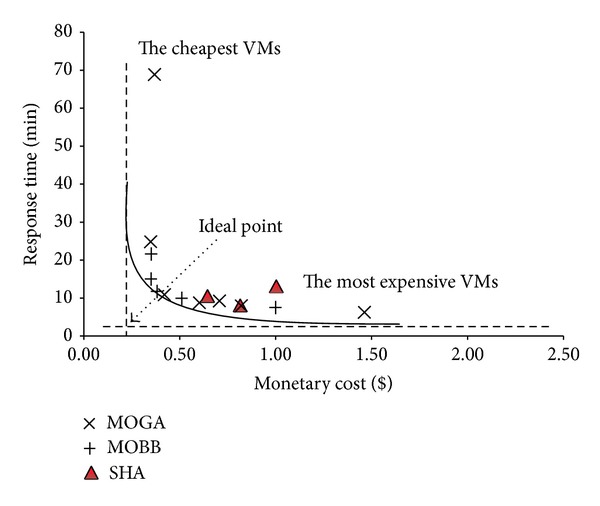
Proposed paretooptimal solutions for workload 2 by SHA, MOBB, and MOGA algorithms.

**Figure 17:**
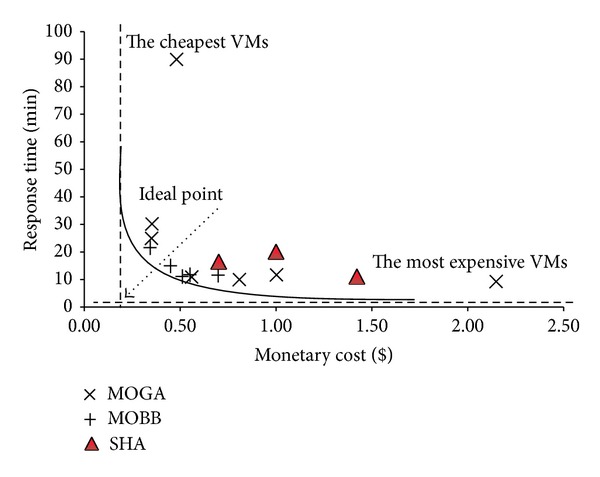
Proposed paretooptimal solutions for workload 3 by SHA, MOBB, and MOGA algorithms.

In Figures 15, 16, and 17 and Tables 10, 11, and 12 we have presented the solutions produced by SHA, MOBB, and MOGA algorithms and the set of proposed VM and network bandwidths, respectively. The solutions with the highest and the cheapest performance VMs are also added to define upper and lower bounds. VMs with the highest configuration capabilities (A7) give the best response time and WMs with the cheapest configurations (XS) give the longest execution time. In this sense, they provide meaningful results to evaluate the quality of solutions provided by MOBB and MOGA.

In the figures, a hypothetical* ideal point* is defined to show the optimal fitness value that can be achieved within the given minimum response time and minimum pricing. The solutions that are chosen from the set of solutions produced by MOBB and MOGA algorithms construct a paretooptimal convex curve where a decision maker can choose any of the solutions according to his/her requirements.* The most expensive VMs* option gives the fastest response time and* the cheapest VMs* option is the most time consuming.

The optimal solutions are produced by MOBB algorithm. MOGA also gives almost the same solutions with faster optimization times than MOBB. The optimization time of MOBB is the longest and it can be prohibitive with workloads having more than 20 queries. In workload 3, MOBB algorithm was 20 times longer than MOGA. The solutions of SHA are slightly above the paretooptimal curve but they are far from the ideal point. Mostly, the most expensive VMs are assigned to VM1 that executes much of the join operations. Workloads 1 and 2 used 100 Mbps network but workload 3 that needs more communication between the VMs tends to use 200 Mbps network.

The solution sets produced by MOBB and MOGA construct a paretooptimal curve and decision makers can choose any of these solutions depending on their needs. The best solutions are near the ideal point. They have fast response times and cheaper monetary costs.

## 7. Conclusions and Future Work

In this paper, we solve the multiobjective optimization problem of Cloud data warehouse query workloads by making use of the elasticity of the virtual resources and alternative query execution plans. We minimize the monetary cost as well as providing fast response times. We formulate the problem and propose three algorithms, namely, simple heuristic (SHA), multiobjective branch-and-bound (MOBB), and multiobjective robust genetic algorithm (MOGA) for the optimization of the problem. To the best of our knowledge, the multiobjective query optimization problem is being solved for the first time with such a method. There are studies that are concerned with the best virtual resource deployment or the minimal monetary cost of workloads in static hardware resources; however, we combine both of these optimization techniques together with alternative query plans and obtain remarkable results as they are presented in our study. It is possible to design and expand the study with additional elastic resources such as I/O bandwidth and dynamic RAMs.

## Figures and Tables

**Algorithm 1:**
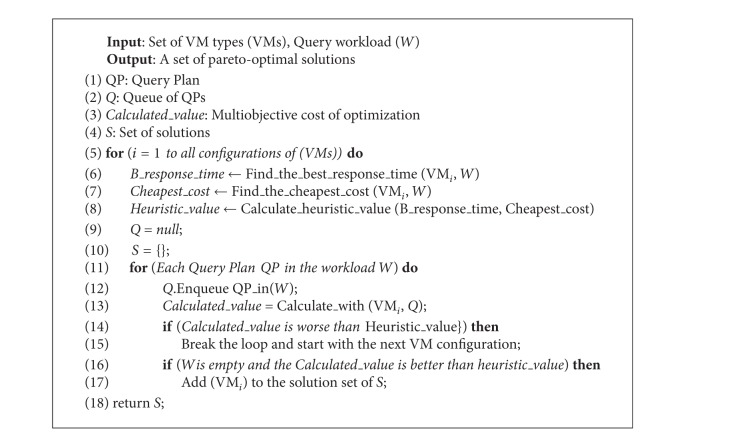
Multiobjective optimization of Cloud database configuration using branch-and-bound.

**Algorithm 2:**
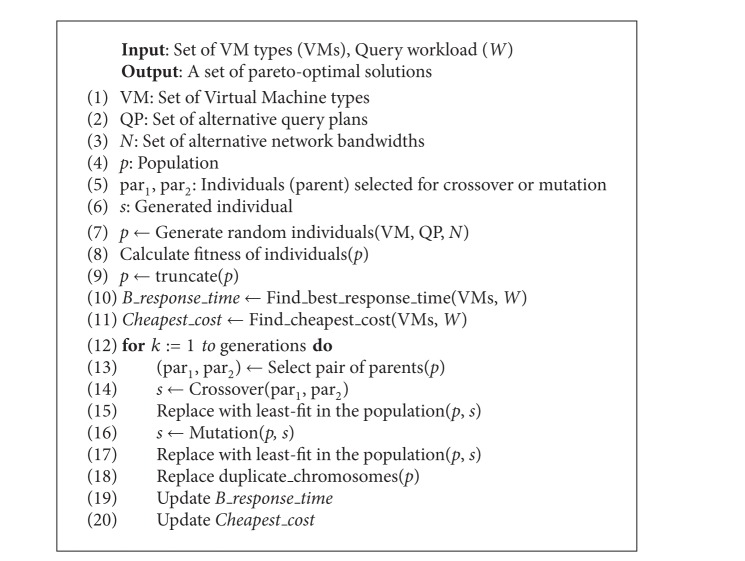
Multiobjective optimization of cloud database configuration using genetic algorithm.

**Table 1:** Response time and monetary costs of TPC-H query 3 (QP_1_) with 6 different virtual resource configurations.

Virtual machine configuration	Network bandwidth	Response time (sec.)	Price ¢
1 × 2 Ghz CPU, 768 MB RAM	10 Mbps	57.0	¢0.048
1 × 2 Ghz CPU, 768 MB RAM	100 Mbps	16.5	¢0.055
1 × 2 Ghz CPU, 768 MB RAM	300 Mbps	15.7	¢0.140
4 × 2 Ghz CPU, 8 GB RAM	10 Mbps	58.2	¢0.404
4 × 2 Ghz CPU, 8 GB RAM	100 Mbps	13.5	¢0.128
4 × 2 Ghz CPU, 8 GB RAM	300 Mbps	13.1	¢0.197

**Table 2:** Response time and monetary costs of TPC-H query 3 (QP_2_) with 6 different virtual resource configurations.

Virtual machine configuration	Network bandwidth	Response time (sec.)	Price ¢
1 × 2 Ghz CPU, 768 MB RAM	10 Mbps	32.5	¢0.027
1 × 2 Ghz CPU, 768 MB RAM	100 Mbps	31.2	¢0.104
1 × 2 Ghz CPU, 768 MB RAM	300 Mbps	29.6	¢0.263
4 × 2 Ghz CPU, 8 GB RAM	10 Mbps	14.6	¢0.101
4 × 2 Ghz CPU, 8 GB RAM	100 Mbps	14.5	¢0.137
4 × 2 Ghz CPU, 8 GB RAM	300 Mbps	14.3	¢0.215

**Table 3:** Parameters used in the cost model.

Symbol	Definition
*T*_I/O_	I/O time for a page
#I/O	Number of page I/O operations
seq_#I/O	Max. number of sequential pages I/O
*T*_CPU_	Time for a CPU instruction
seq_#insts	Max. number of sequential instructions
*T*_MSG_	Time to initiate and receive a message
seq_#msgs	Max. number of sequential messages
T_TR_	Time to transmit a page
seq_#pages	Max. number of sequential pages
#insts	Number of instructions

**Table 4:** Virtual machine prices.

Symbol	Virtual machine configuration	Price
XS	1 GHz CPU, 768 MB RAM	$0.02/hr
S	1.6 GHz CPU, 1.75 GB RAM	$0.06/hr
M	2 × 1.6 GHz CPU, 3.5 GB RAM	$0.12/hr
L	4 × 1.6 GHz CPU, 7 GB RAM	$0.24/hr
XL	8 × 1.6 GHz CPU, 14 GB RAM	$0.48/hr
A_6_	4 × 1.6 GHz CPU, 28 GB RAM	$0.82/hr
A_7_	8 × 1.6 GHz CPU, 56 GB RAM	$1.64/hr

**Table 5:** Cloud database storage prices.

Database size	Price
100 MB	$5.00/mo
1 GB	$9.99/mo
2 GB	$13.99/mo
5 GB	$25.98/mo
10 GB	$45.96/mo
50 GB	$125.88/mo
150 GB	$225.78/mo

**Table 6:** Network bandwidth prices.

Bandwidth	Price
10 Mbps	$0.05/hr
100 Mbps	$0.50/hr
200 Mbps	$1.00/hr

**Table 7:** Execution of multiobjective branch-and-bound algorithm with heuristic values (monetary cost = $1.2 and response time = 50 min.).

State	Monetary cost ($)	Response time (sec.)	Action
〈QP_1_, —, —〉	$0.5	10 min.	Expanded
〈QP_2_, —, —〉	$1.3	25 min.	Pruned
〈QP_3_, —, —〉	$0.5	55 min.	Pruned
〈QP_1_, QP_1_, —〉	$0.9	37 min.	Expanded
〈QP_1_, QP_2_, —〉	$0.9	37 min.	Expanded
〈QP_1_, QP_1_, QP_1_〉	$1.1	42 min.	Solution
〈QP_1_, QP_2_, QP_1_〉	$1.2	45 min.	Solution

**Table 8:** TPC-H queries used in the workloads.

Workload	TPC queries
*W*_1_ (10 queries)	1, 2, 3, 4, 5, 6, 7, 8, 9, 10
*W*_2_ (10 queries)	2, 3, 4, 9, 10, 11, 12, 13, 14, 16
*W*_3_ (15 queries)	1, 3, 5, 6, 7, 8, 9, 10, 11, 12, 13, 18, 20, 22

**Table 9:** Parameter settings for multiobjective genetic algorithm.

MOGA parameter	Value
Population size	40
Number of generations	100
Maximum number of genes to transfer	50%
Minimum number of genes to transfer	10%
Tournament size	10
Mutation ratio	1%

**Table 10:** Proposed paretooptimal VM and network configurations for workload 1.

Solution	Virtual machines	Network type
The most expensive	VM_0_ = A_7_; VM_1_ = A_7_; VM_2_ = A_7_; VM_3_ = A_7_; VM_4_ = A_7_; VM_5_ = A_7_	200 (Mbps)
The cheapest	VM_0_ = XS; VM_1_ = XS; VM_2_ = XS; VM_3_ = XS; VM_4_ = XS; VM_5_ = XS	10 (Mbps)
SHA-1	VM_0_ = L; VM_1_ = A_7_; VM_2_ = A_6_; VM_3_ = XL; VM_4_ = L; VM_5_ = M	10 (Mbps)
SHA-2	VM_0_ = L; VM_1_ = A_7_; VM_2_ = A_6_; VM_3_ = XL; VM_4_ = L; VM_5_ = M	100 (Mbps)
SHA-3	VM_0_ = L; VM_1_ = A_7_; VM_2_ = A_6_; VM_3_ = XL; VM_4_ = L; VM_5_ = M	200 (Mbps)
MOBB-1	VM_0_ = S; VM_1_ = A_7_; VM_2_ = XS; VM_3_ = L; VM_4_ = S; VM_5_ = L	100 (Mbps)
MOBB-2	VM_0_ = XS; VM_1_ = XL; VM_2_ = XL; VM_3_ = S; VM_4_ = S; VM_5_ = XS	100 (Mbps)
MOBB-3	VM_0_ = S; VM_1_ = L; VM_2_ = A_7_; VM_3_ = A_7_; VM_4_ = XS; VM_5_ = L	100 (Mbps)
MOBB-4	VM_0_ = XL; VM_1_ = A_7_; VM_2_ = XL; VM_3_ = A_7_; VM_4_ = XS; VM_5_ = A_6_	100 (Mbps)
MOBB-5	VM_0_ = L; VM_1_ = L; VM_2_ = M; VM_3_ = L; VM_4_ = A_6_; VM_5_ = A_6_	100 (Mbps)
MOGA-1	VM_0_ = XS; VM_1_ = A_7_; VM_2_ = S; VM_3_ = XL; VM_4_ = L; VM_5_ = A_6_	100 (Mbps)
MOGA-2	VM_0_ = S; VM_1_ = XL; VM_2_ = XL; VM_3_ = XL; VM_4_ = S; VM_5_ = XS	100 (Mbps)
MOGA-3	VM_0_ = XS; VM_1_ = XL; VM_2_ = XL; VM_3_ = XL; VM_4_ = S; VM_5_ = S	100 (Mbps)
MOGA-4	VM_0_ = L; VM_1_ = S; VM_2_ = XS; VM_3_ = XL; VM_4_ = A_7_; VM_5_ = L	100 (Mbps)
MOGA-5	VM_0_ = XS; VM_1_ = S; VM_2_ = XS; VM_3_ = A_7_; VM_4_ = A_6_; VM_5_ = XS	100 (Mbps)

**Table 11:** Proposed paretooptimal VM and network configurations for workload 2.

Solution	Virtual machines	Network type
The most expensive	VM_0_ = A_7_; VM_1_ = A_7_; VM_2_ = A_7_; VM_3_ = A_7_; VM_4_ = A_7_; VM_5_ = A_7_	200 (Mbps)
The cheapest	VM_0_ = XS; VM_1_ = XS; VM_2_ = XS; VM_3_ = XS; VM_4_ = XS; VM_5_ = XS	10 (Mbps)
SHA-1	VM_0_ = L; VM_1_ = A_7_; VM_2_ = A_6_; VM_3_ = XL; VM_4_ = L; VM_5_ = M	10 (Mbps)
SHA-2	VM_0_ = L; VM_1_ = A_7_; VM_2_ = A_6_; VM_3_ = XL; VM_4_ = L; VM_5_ = M	100 (Mbps)
SHA-3	VM_0_ = L; VM_1_ = A_7_; VM_2_ = A_6_; VM_3_ = XL; VM_4_ = L; VM_5_ = M	200 (Mbps)
MOBB-1	VM_0_ = XS; VM_1_ = XL; VM_2_ = S; VM_3_ = XL; VM_4_ = XL; VM_5_ = XS	100 (Mbps)
MOBB-2	VM_0_ = XS; VM_1_ = XL; VM_2_ = XL; VM_3_ = XL; VM_4_ = XL; VM_5_ = XS	100 (Mbps)
MOBB-3	VM_0_ = S; VM_1_ = XL; VM_2_ = S; VM_3_ = XL; VM_4_ = XL; VM_5_ = L	100 (Mbps)
MOBB-4	VM_0_ = XL; VM_1_ = M; VM_2_ = L; VM_3_ = XL; VM_4_ = XS; VM_5_ = A_7_	100 (Mbps)
MOBB-5	VM_0_ = XS; VM_1_ = XL; VM_2_ = S; VM_3_ = XL; VM_4_ = XL; VM_5_ = S	100 (Mbps)
MOGA-1	VM_0_ = XS; VM_1_ = XL; VM_2_ = XS; VM_3_ = XL; VM_4_ = XL; VM_5_ = S	100 (Mbps)
MOGA-2	VM_0_ = L; VM_1_ = XL; VM_2_ = L; VM_3_ = XL; VM_4_ = M; VM_5_ = S	100 (Mbps)
MOGA-3	VM_0_ = M; VM_1_ = A_7_; VM_2_ = S; VM_3_ = A_7_; VM_4_ = XL; VM_5_ = M	100 (Mbps)
MOGA-4	VM_0_ = S; VM_1_ = XL; VM_2_ = M; VM_3_ = A_7_; VM_4_ = XL; VM_5_ = S	100 (Mbps)
MOGA-5	VM_0_ = S; VM_1_ = A_7_; VM_2_ = XS; VM_3_ = XL; VM_4_ = XL; VM_5_ = S	100 (Mbps)

**Table 12:** Proposed paretooptimal VM and network configurations for workload 3.

Solution	Virtual machines	Network type
The most expensive	VM_0_ = A_7_; VM_1_ = A_7_; VM_2_ = A_7_; VM_3_ = A_7_; VM_4_ = A_7_; VM_5_ = A_7_	200 (Mbps)
The cheapest	VM_0_ = XS; VM_1_ = XS; VM_2_ = XS; VM_3_ = XS; VM_4_ = XS; VM_5_ = XS	10 (Mbps)
SHA-1	VM_0_ = L; VM_1_ = A_7_; VM_2_ = A_6_; VM_3_ = XL; VM_4_ = L; VM_5_ = M	10 (Mbps)
SHA-2	VM_0_ = L; VM_1_ = A_7_; VM_2_ = A_6_; VM_3_ = XL; VM_4_ = L; VM_5_ = M	100 (Mbps)
SHA-3	VM_0_ = L; VM_1_ = A_7_; VM_2_ = A_6_; VM_3_ = XL; VM_4_ = L; VM_5_ = M	200 (Mbps)
MOBB-1	VM_0_ = S; VM_1_ = XL; VM_2_ = L; VM_3_ = XL; VM_4_ = M; VM_5_ = M	200 (Mbps)
MOBB-2	VM_0_ = XS; VM_1_ = XL; VM_2_ = XL; VM_3_ = XL; VM_4_ = XL; VM_5_ = S	200 (Mbps)
MOBB-3	VM_0_ = L; VM_1_ = A_6_; VM_2_ = L; VM_3_ = M; VM_4_ = M; VM_5_ = XS	200 (Mbps)
MOBB-4	VM_0_ = A_7_; VM_1_ = M; VM_2_ = A_6_; VM_3_ = XL; VM_4_ = A_6_; VM_5_ = A_6_	200 (Mbps)
MOBB-5	VM_0_ = A_6_; VM_1_ = A_7_; VM_2_ = L; VM_3_ = S; VM_4_ = L; VM_5_ = A_7_	200 (Mbps)
MOGA-1	VM_0_ = M; VM_1_ = M; VM_2_ = A_6_; VM_3_ = A_7_; VM_4_ = A_6_; VM_5_ = L	200 (Mbps)
MOGA-2	VM_0_ = A_6_; VM_1_ = A_7_; VM_2_ = XS; VM_3_ = M; VM_4_ = A_6_; VM_5_ = XL	200 (Mbps)
MOGA-3	VM_0_ = XS; VM_1_ = XL; VM_2_ = L; VM_3_ = XS; VM_4_ = L; VM_5_ = L	200 (Mbps)
MOGA-4	VM_0_ = S; VM_1_ = L; VM_2_ = S; VM_3_ = A_7_; VM_4_ = A_7_; VM_5_ = XS	200 (Mbps)
MOGA-5	VM_0_ = M; VM_1_ = A_7_; VM_2_ = M; VM_3_ = A_7_; VM_4_ = L; VM_5_ = S	200 (Mbps)

## Appendix

### A. Alternative Query Execution Plans for TPC-H Query Q3

TPC-H Q3 statement in accordance with the query execution plan 1 where all of the tables are shipped to query issuing node:

select top 10 l_orderkey,…, o_shippriority
from [vm_2_].customer c, [vm_3_].orders o, [vm_1_].lineitem l,
where c.c_mktsegment = “building”
and c.c_custkey = o.o_custkey
and l.l_orderkey = o.o_orderkey
and o.o_orderdate < “1995-03-15”
and l.l_shipdate > “1995-03-15”
group by l.l_orderkey, o.o_orderdate, o.o_shippriority
order by revenue desc, o.o_orderdate.

TPC-H Q3 statement in accordance with query execution plan 2 where customer and orders tables are joined at virtual machine 3 and the resulting tuples are shipped to virtual machine 2 to join with lineitem table:

select top 10 l_orderkey,…, o_shippriority
from
openquery ([vm_3_]. “select o_orderdate, o_shippriority, o_orderkey
from [vm_2_].customer c, [vm_3_].orders o
where c.c_mktsegment = “building”
and c.c_custkey = o.o_custkey
and o.o_orderdate < “1995-03-15”
group by o.o_orderdate, o.o_shippriority, o_orderkey
order by o.o_orderdate) remote1, [vm_1_].lineitem l
where and l.l_orderkey = remote1.o_orderkey
and l.l_shipdate > “1995-03-15”
group by l.l_orderkey, remote1.o_orderdate,
remote1.o_shippriority
order by reveneu desc”.
